# Development and application of a dual ERA method for the detection of Feline Calicivirus and Feline Herpesvirus Type I

**DOI:** 10.1186/s12985-023-02020-3

**Published:** 2023-04-05

**Authors:** Bo Chen, Haoyang Zhang, Hanhong Wang, Shoujun Li, Pei Zhou

**Affiliations:** 1 Guangdong Provincial Pet Engineering Technology Research Center, College of Veterinary Medicine, South China Agricultural University, Guangzhou, 510642 Guangdong Province People’s Republic of China; 2 College of Veterinary Medicine, South China Agricultural University, Guangzhou, 510642 Guangdong Province People’s Republic of China

**Keywords:** ERA, FCV, FHV-1, Dual detection, Clinical detection

## Abstract

Feline calicivirus (FCV) and feline herpesvirus type I (FHV-1) are the most common viral pathogens responsible for cat respiratory diseases, and coinfection with these two pathogens is often found. In veterinary clinics, the main diagnostic methods for FCV and FHV-1 are test strips and polymerase chain reaction (PCR). However, the sensitivity of test strips are not sufficient, and PCR is time-consuming. Therefore, developing a rapid and high-performance clinical diagnostic test is imperative for the prevention and treatment of these diseases. Enzymatic recombinase amplification (ERA) is an automated isothermal nucleic acid amplification technique that maintains a constant temperature, and is both rapid and highly accurate. In this study, a dual ERA method was developed using the Exo probe for a differential detection of FCV and FHV-1. This dual ERA method demonstrated high performance with the detection limit of 10^1^ copies for both viruses, and no cross-reactions with feline parvovirus virus and F81 cells. To test the utility of the method for clinical applications, 50 nasopharyngeal swabs from cats with respiratory symptoms were collected and tested. The positive rates of FCV and FHV-1 were 40% (20/50, 95% confidence interval [CI], 26.4 to 54.8%) and 14% (7/50, 95% CI, 5.8 to 26.7%), respectively. The rate of coinfection with FCV and FHV-1 was 10% (5/50, 95% CI, 3.3 to 21.8%). These results were in agreement with those found using quantitative real-time PCR. Therefore, this dual ERA method is a novel and efficient clinical diagnostic tool for FCV and FHV-1 detection.

## Introduction

Feline upper respiratory tract disease (FURTD) is a common disease in cats and is mostly caused by infectious pathogens [1]. Feline calicivirus (FCV) and feline herpesvirus type I (FHV-1) are the most common viral pathogens of FURTD [2]. Even, coinfection with FCV and FHV-1 is often found in clinical cases [3].

FCV is a single-stranded positive-sense nonenveloped RNA virus that is a member of the genus *Vesivirus*, family *Caliciviridae*. FCV is highly infectious among feline populations [4]. In addition to causing upper respiratory symptoms in cats, it usually also causes oral ulcers in cats. However, virulent systemic FCV (VS-FCV) can cause severe and acute virulent systemic disease (VSD) in cats, and it even has a significant effect on vaccinated adult cats, including an increased risk of death [5, 6]. Moreover, while FCV vaccination can protect cats from severe symptoms, it cannot completely prevent infection with the virus or viral shedding [7, 8].

FHV-1 is a double-stranded enveloped DNA virus belonging to the family *Herpesviridae*, subfamily *alpha-herpesvirus*, and genus *varicella virus*. FHV-1 causes respiratory symptoms and conjunctivitis in cats [9, 10]. Kittens and immunocompromised adult cats are susceptible to FHV-1. After infection, the virus can be detected in the mouth, nose and eyes of cats [11, 12]. After treatment, the virus lives in the trigeminal nerve of recovered cats and sheds discontinuously under stress situations [11].

Infection with FCV or FHV-1 is clinically common, and the typical features of these two viruses are similar, including eye and nasal secretions, sneezing, and conjunctivitis [13, 14]. Asymptomatic carriers of the two viruses have long-term viral shedding; therefore, FCV or FHV-1 detection is essential in veterinary clinics. Currently, the main diagnostic methods for FCV and FHV-1 are test strips and polymerase chain reaction (PCR). However, the sensitivity of test strips are not sufficient [15–17], and PCR is time-consuming. Therefore, developing a rapid and high-performance clinical diagnostic test is imperative for preventing and treating these diseases.

ERA is an isothermal nucleic acid amplification technology developed by GenDx Biotech, which is a modified version of recombinase polymerase amplification (RPA) [18]. The basic principle is to use recombinases and primers to form protein-DNA complexes that search for homologous sequences in double-stranded DNA and promote strand displacement at the homologous sites. Then, a single-stranded DNA binding protein (SSB) binds to the replaced template strand, and finally initiates the ERA reaction, thereby achieving exponential amplification of the target gene. Based on this method and the use of probes, the amplification process can be monitored in real time by designing probes.

In this study, a dual ERA method for the simultaneous detection of FCV and FHV-1 was developed.

## Materials and methods

### Viruses, cells and clinical samples

The viruses, FCV, FHV-1 and feline parvovirus virus (FPV), were previously isolated and stored in our laboratory. FCV and FHV-1 were cultured using feline kidney F81 cells, which were cultured in Dulbecco’s modified Eagle’s medium (DMEM) plus 10% fetal bovine serum (FBS). Fifty nasopharyngeal swabs of cats with respiratory symptoms were collected from pet hospitals in Guangzhou.

### Nucleic acid extraction

According to the manufacturer’s instructions, viral nucleic acids were extracted using the RaPure Viral RNA/DNA Kit (Magen, China), and F81 DNA was extracted using the TIANamp Genomic DNA Kit (Tiangen, China). The reverse transcription reaction was performed to synthesize first-strand cDNA following the manufacturer’s instructions (Vazyme, China). Eventually, the samples were stored in a -80 ℃ freezer.

### Standard recombinant plasmid preparations

pMD-FCV-F/R and pMD-FHV-1-F/R are primers designed from the genetically conserved regions of FCV VP1 and FHV-1 gB with Primer Premier 5.0 software (Table 1). The composition of the PCR mixture included 10 µL of 2× Taq PCR Star Mix (GenStar, China), 1 µL of each of the F/R primers (10 µmol/L), 1 µL of sample DNA/cDNA, and 7 µL of double-distilled water (ddH_2_O). FCV and FHV-1 were amplified using the following procedure: predenaturation at 95 °C for 3 min, followed by 35 cycles of denaturation at 95 °C for 25 s, annealing at 54 or 55 °C for 25 s, and extension at 72 °C for 30 s, with a final extension step at 72 °C for 5 min. The generated fragments were resolved on a 1.5% (W/V) agarose gel. Then, PCR products were purified using FastPure Gel DNA (Vazyme, China) and cloned into a pMD18-T vector (Takara, Japan), which resulted in the construction of two recombinant plasmids named pMD18T-FCV and pMD18T-FHV-1. Plasmids were extracted from bacterial suspensions containing accurate gene sequences using the EndoFree Mini Plasmid Kit II (Tiangen, China). The concentrations of the recombinant plasmids pMD- FCV and pMD- FHV-1 were measured, and then the numbers of copies were calculated.

### ERA primer and probe design

ERA probes and three pairs of corresponding primers were designed within the highly conserved fragments of the FCV VP1 gene and FHV-1 gB gene using Primer Premier 5.0 software (Table 2). The specificity of the primers and probes was evaluated by nucleotides BLAST of the National Center for Biotechnology Information (NCBI) to ensure that no results with strong matches to other feline viral sequences were found. The above primers and probes were synthesized at Sangon Biotech Co. Ltd (Shanghai, China).

### ERA primer screening

To determine the validity of the primers, the recombinant plasmids pMD-FCV and pMD-FHV-1 were used as templates, and the designed forward and reverse primers were paired and subjected to PCR at an annealing temperature of 55 °C.

A fluorescent-type nucleic acid amplification kit (GenDx, China) was used to screen the primers using the ERA method. The procedure was as follows: 20 µL of deliquescent agent, 1.05 µL of each forward and reverse primer (10 µmol/L) for FHV-1 and FCV, 0.3 µL of FHV-Probe, 0.3 µL of FCV-Probe, 2 µL of template, 2 µL of activator, and ddH_2_O were made up to 50 µL. Then, the mixture was rapidly placed into the real-time fluorescence quantitative PCR instrument. The fluorescence information of FAM/ROX was collected every 15 s, at 37 °C. Tests were performed in triplicate to obtain the average cycle threshold (Ct) values of the primers and thus to screen for the best-amplified primer pairs.

### Optimization of the dual ERA method

The probe concentration, primer concentration, and reaction temperature of the ERA reaction mixture all have a significant impact on the amplification effect. According to the instructions of GenDx (Suzhou, China), the reaction temperature ranged from 37 to 42 °C, the volume of the probe solution (10 µmol/L) was 0.6 µL, and the volume of the primer solution (10 µmol/L) was 2.1 µL. Each factor was divided into three levels to design the L9 (3^4^) orthogonal test protocol for the dual ERA method (Table 3). The optimal amplification primer pair was utilized for this optimized protocol, and fluorescence detection was performed every 15 s for a total of 80 times. Tests were performed in triplicate to obtain the average Ct values. Minitab software was used to determine the optimal level for each factor.

### Sensitivity analysis

The copy number of pMD-FCV and pMD-FHV-1 was calculated by the formula: amount (copies/µL) = 6 × 10^23^ (copies/mol)×concentration (g/µL)/MW (g/mol). For analyze sensitivity, the recombinant plasmids were diluted from 10^1^ to 10^6^ copies/µL and subsequently tested by the ERA method.

### Specificity analysis

​ DNA or cDNA from FPV and F81 cells was used for ERA specificity analysis to eliminate cross-reactions in the ERA method with other feline viruses or genomes.

### Clinical sample detection

Fifty nasopharyngeal swabs from cats with respiratory symptoms (all vaccinated) were collected from pet hospitals in Guangzhou to evaluate the performance of the dual ERA method in the detection of FCV and FHV-1 in clinical samples, and the results were compared with quantitative real-time PCR (qRT‒PCR) [19]. All samples were stored in a -80 °C freezer.

## Results

### Screening of the optimal primer for the ERA assay

**Fig. 1:**
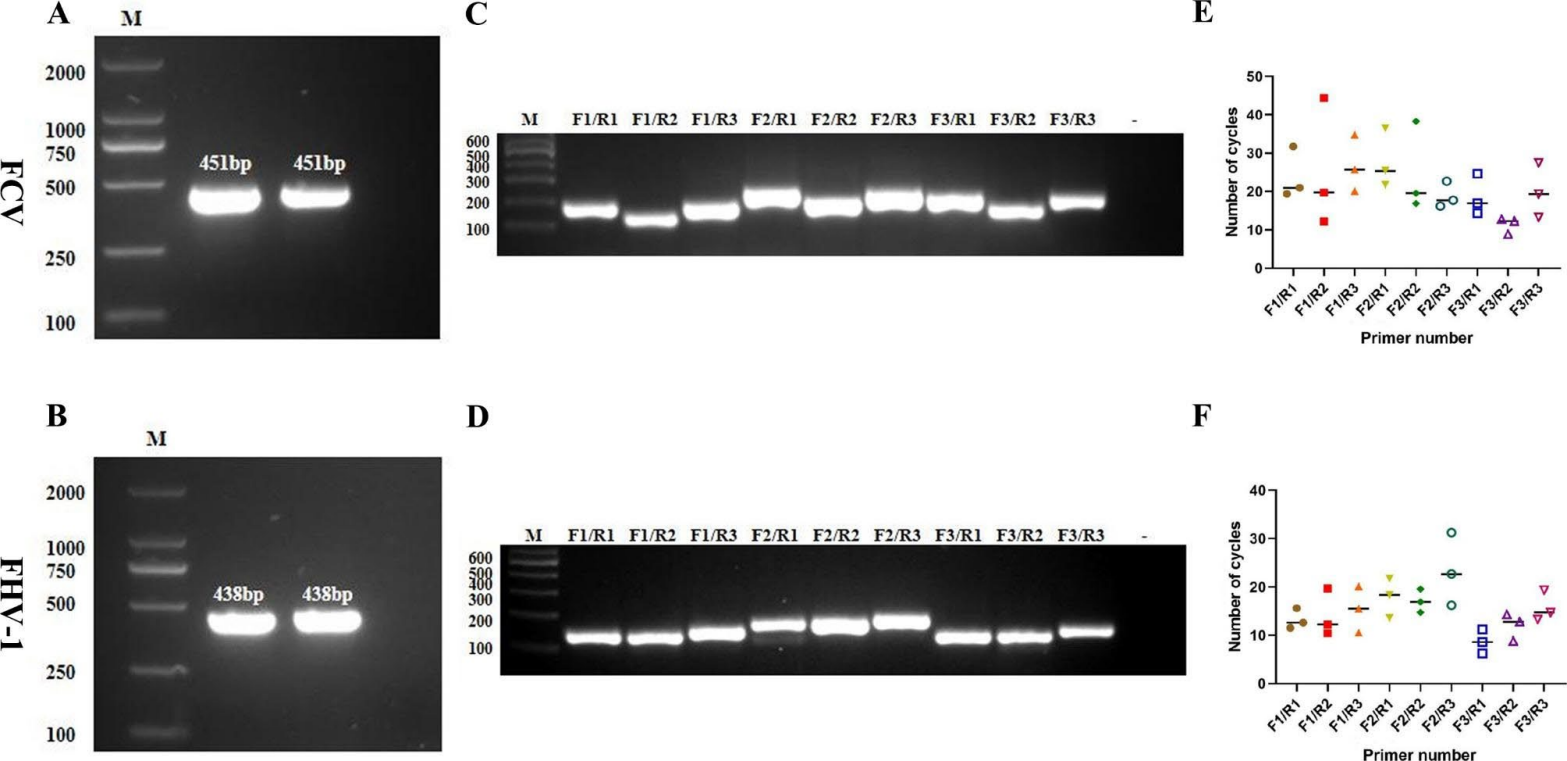
Amplification results for the FCV and FHV-1 target fragments. The amplified PCR products were electrophoresed in a 1.5% (W/V) agarose gel for 30 min at 120 V. **(A)** Lanes 1 and 2 are the target fragments required for the construction of pMD-FCV. **(B)** Lanes 1 and 2 are the target fragments required for the construction of pMD-FHV-1. Screening of optimal FCV **(C, E)** and FHV-1 **(D, F)** primer pairs

The target fragments of FCV and FHV-1 were amplified using PCR (Fig. 1A, B). The PCR products were retrieved to construct pMD-FCV and pMD-FHV-1 plasmids, whose sequences were verified using sequencing (data not shown).

The optimal primers of the ERA assay were screened by a series of PCR tests. First, each primer pair has demonstrated efficient PCR amplification (Fig. 1C, D). Second, each primer pair was tested using the ERA method. To visualize the performance of the primers, the Ct value at which the fluorescence intensity reached the positive threshold was plotted on the Y-axis, while the serial number of each primer pair was plotted on the X-axis. Under these conditions, the lower the Ct and SD values were, the better the amplification efficiency and stability. Finally, FCV-F3/R2 and FHV-F3/R1 were shown to have the lowest and most stabilized Ct values (Fig. 1E, F). In summary, the primer pairs FCV-F3/R2 and FHV-F3/R1 were chosen as the optimal primer pairs for the dual ERA method.

### Orthogonal analysis and establishment of the dual ERA method

There were significant differences in the amplification effect due to the different combinations of temperature, probe ratio and primer ratio. The R value for each factor represents its effect on the test. According to the R value in the L9 analysis (Tables 4 and 5), the influence of each factor on the Ct value from largest to smallest is temperature (A) > FHV: FCV primer volume (B) > FHV: FCV probe volume (C). The lowest average Ct value for the different levels of each factor was chosen as the primary reference from which to derive the optimal level for each factor. The optimal level for the dual ERA method of FCV was A_3_B_1_C_2_ (Table 5); the optimal level for the dual ERA method of FHV-1 was A_3_B_3_C_2_ (Table 5). To ensure efficient amplification of both pathogens under the same conditions, we need to find the optimal condition, i.e., the difference between the average CT values of the two pathogens should be minimized. This optimal level is A_1_B_1_C_1_ (Table 5).

In the final determination of the optimal level for this method, the vital reference element was the frequency of optimal levels. The higher the frequency of the optimal level is, the better the integration advantage. In the selection of temperature (A), A_3_ had two optimal levels. In the selection of the FHV: FCV primer volume (B), B_1_ had two optimal levels that were large. In the selection of the FHV: FCV probe volume (C), C_2_ had two optimal levels. Therefore, we established the optimal reaction conditions for the dual ERA as A_3_B_1_C_2_ (Table 6).

### Sensitivity analysis and specificity analysis

**Fig. 2:**
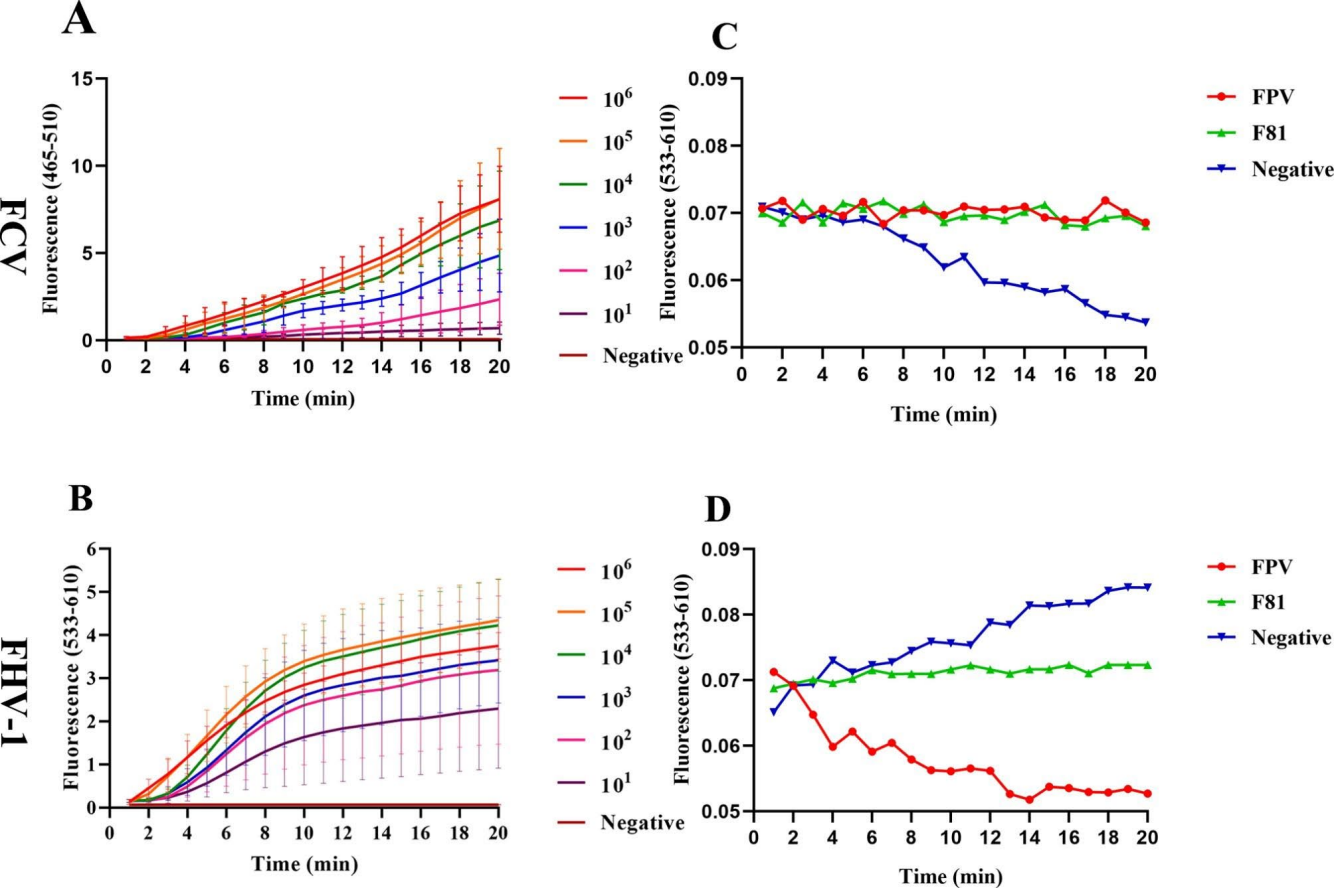
Sensitivity and specificity analysis of the dual ERA methods. The negative control was ddH_2_O. **(A, B)** Detection limits of pMD-FCV and pMD-FHV-1. **(C, D)** Specificity analysis of ERA-FCV and ERA-FHV-1.

For sensitivity, copy numbers ranging from 10^6^ to 10^1^ of the plasmids were amplified by the established dual ERA procedure. The results showed that the lower limit of detection was 10^1^ copies for both viruses (Fig. 2A, B).

For specificity, only FCV and FHV-1 were amplified by the established dual ERA method. Notably, FPV and F81 cells had no cross-reaction (Fig. 2C, D), which indicates that the established dual ERA has excellent specificity.

### Detection of clinical samples by the dual ERA method

Fifty nasopharyngeal swabs from cats with respiratory symptoms were collected for testing using the dual ERA method (Table 7). In total, 44% (22/50, 95% confidence interval [CI], 30 to 58.7%) samples were positive. Among them, 40% (20/50) of the samples were positive for FCV, and 14% (7/50) of the samples were FHV-1 positive. Moreover, 10% (5/50) of the samples were coinfected with FCV and FHV-1. All of the positive and negative samples were verified by qRT‒PCR.

## Discussion

At present, the proportion of pet cats in pet families is rising daily, and the number has exceeded that of dogs [20]. FCV and FHV-1 are the two most common feline respiratory tract viruses, and they threaten the health of cats. In particular, there is high morbidity in multicats and kittens families [21]. Even though a vaccine has been developed, it seems that cats have less immunity to the virus than was expected [22]. Therefore, the development of a rapid and simultaneous detection method for FCV and FHV-1 plays an indispensable role in the prevention and control of these two viral pathogens.

As a modified version of RPA, ERA can be carried out at a constant temperature without the need for thermocycles [18]. In addition, ERA has significant advantages over traditional PCR and other isothermal nucleic acid amplification methods in terms of time and portability [23]. Because of these characteristics, this technology has been applied in the large-scale detection of various pathogens, such as viruses, bacteria, fungi, mycoplasma and Plasmodium falciparum [24–28]. ERA has been combined with different technologies to satisfy different detection needs. For example, reverse transcription ERA (RT-ERA) uses modified RNA reverse transcriptase to reverse transcribe RNA; at the same time, DNA recombinase starts DNA synthesis using cDNA as a template, which makes detection simpler and more reliable [29]. ERA combined with the lateral flow dipstick (LFD) technique can be used to quickly identify the pathogen [30, 31]. Even combining ERA with the CRISPR‒Cas12a system can produce results visible to the naked eye under LED blue light [32]. Furthermore, ERA combined with the Exo probe can detect multiple pathogens in real time, achieve excellent specificity and sensitivity [33], and offer better speed, portability, and accessibility than qRT‒PCR [34]. Obviously, an important advantage of ERA is that it is a point-of-care (POC) detection method. Of course, there are still some limitations that we should be aware of. First, unfortunately, certain requirements are necessary for ERA testing conditions and personnel, such as DNA/RNA extraction and reverse transcription of samples must be carried out in steps before nucleic acid testing. Second, the reaction speed of ERA is quite fast, and the fluorescence value reaches a plateau in a short time. Therefore, after adding all the reagent components, the change in fluorescence value should be measured quickly. Third, after adding the activator, the ERA reaction will be amplified at room temperature, so at present it can only be qualitatively detected for the pathogen in the test sample, and quantitative detection has not yet been achieved. Finally, after the amplification is completed, it is necessary to inactivate the nucleic acid in the tube by heating at high temperature to avoid nucleic acid contamination.

In this study, a dual ERA method for simultaneous detection of FCV and FHV-1 was developed by designing probes and primers targeting the conserved regions of the FCV VP1 gene and FHV-1 gB gene. The design of primers and probes is the key step for dual ERA. According to the principle of ERA primer design, the primer length was between 30 and 35 bp, and the use of degenerate bases was minimized; The required probe length for ERA was 46–52 bp, where the fluorophore was at least 30 nucleotides away from the 5’ end and the quencher was at least 15 nucleotides away from the 3’ end. The fluorophore and quencher were prelinked to the T-base, and they were linked by tetrahydrofuran (THF) with a blocking group inserted at the 3’ end. After screening and optimization, the lower limit of detection of the dual ERA method for both viruses was 10^1^ copies, and there was no cross-reaction with FPV and F81 cells. Wang *et al*. developed an RPA method targeting the thymidine kinase (TK) gene of FHV-1 with a lower limit of detection of 10^2^ copies [35]. Huang *et al*. developed an RPA method targeting the FCV ORF1 gene with a lower limit of detection of 5.5 copies [36]. The sensitivity of the dual ERA method developed in this study is similar to or higher than that of the above methods. FCV (40%, 20/50) and FHV-1 (14%, 7/50) were detected in the vaccinated cats, which is consistent with the findings of previous studies [37], and this has raised commercial vaccine protection concerns in China. In addition, 10% (5/50, 95% CI, 3.3 to 21.8%) coinfection of FCV and FHV-1 was detected in this study, which is consistent with the results in previous reports [3] and indicates that the treatment strategy should take into account the scenario of coinfection with both of these viruses. Moreover, dual ERA detection results were consistent with those from qRT‒PCR assays used in clinical applications, indicating the reliability and validity of this novel dual ERA method.

In summary, a high-performance, rapid and reliable dual ERA detection method was developed for the simultaneous detection of FCV and FHV-1, which could be developed as a novel efficient clinical diagnostic tool for FCV and FHV-1 detection.

**Table 1:** Primers for pMD-FCV and pMD-FHV-1 recombinant plasmids

Primer^a^	Primer sequences (5’-3’)	Fragment length (bp)
pMD-FCV-F	GTCNAAHARGGAYCTCACCC	451
pMD-FCV-R	YTCBCKMAAGATTTCAGHCC	
pMD-FHV-1-F	TCCCGTGAAAGTTCAAGAGAT	438
pMD-FHV-1-R	AGTTGTAATCGCGTATCCAAG	

^a^F, forward primer; R, reverse primer.

**Table 2:** Primers and probes for the dual ERA method

Primer^a^	Primer sequences (5’-3’)^b^	Segment length (bp)
FCV-Probe	CTCTTCGCCGBCACCTDWCDYTRDCWGGRCAG (FAM-dT)(THF)(BHQ1-dT)CARGCCATCAGAGCC	
FHV-Probe	TGCTGCTGGATTTCACCACTCTGGGACCTC (ROX- dT)(THF)(BHQ2-dT)AAATTGCATCGTAGA	
FCV-F1	TGTGGTAACYGTTAAYTCRGTGTTTGATTTRGC	173
FCV-R1	RAARGARGTYTCATCCATCCAGTGYCKYAR	
FCV-F2	TCAACGAYARTGTNAGRYTGGATGAACTACC	135
FCV-R2	GNABYTTGTCRGGGRCAGTSAGCACATCATA	
FCV-F3	TGAACTACCCGCCAATCAACATGTGGTAACC	137
FCV-R3	GANGTYTCATCCATCCAGTGYCKCAACATSG	
FHV-F1	GATGGCACACCACCAATGAAACATACACAAAGA	188
FHV-R1	ACGTCACCAGTGGAGATAGCAAATGAGTCA	
FHV-F2	TCTGAAACCCTCCAAGTTCAACACTCCAGAG	195
FHV-R2	ACGTCACCAGTGGAGATAGCAAATGAGTCATA	
FHV-F3	CACACACCAATGAAACATACACAAAGATCGG	167
FHV-R3	GAGACATGTGAATCACGTCACCAGTGGAGAT	

^a^F, forward primer; R, reverse primer.

^b^FAM, 6-Carboxy fluorescein; BHQ, black hole quencher.

**Table 3:** L9 (3^4^) orthogonal test table for the dual ERA method

Test No.	Factors					
	(A) Temperature (℃)		(B) FHV:FCV Primer volume (µL)		(C) FHV:FCV Probe volume (µL)	
1	A_1_	37	B_1_	0.8: 1.3	C_1_	0.2: 0.4
2	A_1_	37	B_2_	1.05: 1.05	C_2_	0.3: 0.3
3	A_1_	37	B_3_	1.3: 0.8	C_3_	0.4: 0.2
4	A_2_	39	B_1_	0.8: 1.3	C_2_	0.3: 0.3
5	A_2_	39	B_2_	1.05: 1.05	C_3_	0.4: 0.2
6	A_2_	39	B_3_	1.3: 0.8	C_1_	0.2: 0.4
7	A_3_	42	B_1_	0.8: 1.3	C_3_	0.4: 0.2
8	A_3_	42	B_2_	1.05: 1.05	C_1_	0.2: 0.4
9	A_3_	42	B_3_	1.3: 0.8	C_2_	0.3: 0.3

**Table 4:** L9 (3^4^) test results

Test no.	Parameters			FCV CT value	FHV-1 Ct Value	Ct value difference
	A	B	C			
1	A_1_	B_1_	C_1_	15.95	14.64	1.31
2	A_1_	B_2_	C_2_	16.48	13.42	3.06
3	A_1_	B_3_	C_3_	20.16	12.63	7.53
4	A_2_	B_1_	C_2_	13.56	9.64	3.92
5	A_2_	B_2_	C_3_	14.75	9.72	5.03
6	A_2_	B_3_	C_1_	16.40	10.39	6.01
7	A_3_	B_1_	C_3_	10.62	8.72	1.90
8	A_3_	B_2_	C_1_	13.01	7.53	5.48
9	A_3_	B_3_	C_2_	15.12	7.51	7.61

**Table 5:** Analysis of L9 (3^4^) test results

	FCV CT value			FHV-1 Ct Value			Ct value difference		
	A	B	C	A	B	C	A	B	C
K_1_	52.59^a^	40.13	45.36	40.69	33.00	32.56	11.90	7.13	12.80
K_2_	44.71	44.24	45.16	29.75	30.67	30.57	14.96	13.57	14.59
K_3_	38.75	51.68	45.53	23.76	30.53	31.07	14.99	21.15	14.46
k_1_	17.53^b^	13.38	15.12	13.56	11.00	10.85	3.97	2.38	4.27
k_2_	14.90	14.75	15.05	9.92	10.22	10.19	4.99	4.52	4.86
k_3_	12.92	17.23	15.18	7.92	10.18	10.36	5.00	7.05	4.82
R	4.61^c^	3.85	0.13	5.64	0.82	0.66	1.03	4.67	0.59
Optimum	A_3_	B_1_	C_2_	A_3_	B_3_	C_2_	A_1_	B_1_	C_1_

a $${K}_{i}^{A}=\sum the amount of CT \text{v}\text{a}\text{l}\text{u}\text{e} at {A}_{i}$$.

b $${k}_{i}^{A}={K}_{i}^{A}/3$$.

c $${R}_{i}^{A}=max\left\{{K}_{i}^{A}\right\}-min\left\{{K}_{i}^{A}\right\}$$.

**Table 6:** The dual ERA optimal reaction conditions

Parameters	Amplification conditions
Temperature /℃	42
FHV-1 probe volume/µL	0.3
FHV-1 primer volume/µL	1.6
FCV probe volume/µL	0.3
FCV primer volume/µL	2.6
Activator/µL	2
Deliquescent agent/µL	20
Template volume /µL	2
ddH_2_O volume /µL	21.2

**Table 7:** Results of the dual ERA method for clinical samples

Viral pathogens	Dual ERA	
	Positive/Total	% (95% CI)
FCV	20/50	40% (26.4 to 54.8%)
FHV-1	7/50	14% (5.8 to 26.7%)
Coinfection	5/50	10% (3.3 to 21.8%)

## Data Availability

The datasets used and/or analyzed during the current study are available from the corresponding author on reasonable request.
